# Pharmacological and Epigenetic Regulators of NLRP3 Inflammasome Activation in Alzheimer’s Disease

**DOI:** 10.3390/ph14111187

**Published:** 2021-11-20

**Authors:** Francesca La Rosa, Roberta Mancuso, Simone Agostini, Federica Piancone, Ivana Marventano, Marina Saresella, Ambra Hernis, Chiara Fenoglio, Daniela Galimberti, Elio Scarpini, Mario Clerici

**Affiliations:** 1 IRCCS Fondazione Don C. Gnocchi, ONLUS, 20148 Milan, Italy; rmancuso@dongnocchi.it (R.M.); sagostini@dongnocchi.it (S.A.); fpiancone@dongnocchi.it (F.P.); imarventano@dongnocchi.it (I.M.); msaresella@dongnocchi.it (M.S.); ahernis@dongnocchi.it (A.H.); mario.clerici@unimi.it (M.C.); 2 Department of Pathophysiology and Transplantation, University of Milan, 20122 Milan, Italy; chiara.fenoglio@unimi.it; 3 Fondazione Cà Granda, IRCCS Ospedale Maggiore Policlinico, 20122 Milan, Italy; daniela.galimberti@unimi.it (D.G.); elio.scarpini@unimi.it (E.S.); 4 Department of Biomedical, Surgical and Dental Sciences, University of Milan, 20100 Milan, Italy

**Keywords:** Alzheimer’s disease, D4T, miRNAs, NLRP3 inflammasome

## Abstract

Activation of the NLRP3 inflammasome complex results in the production of IL-18, Caspase-1 and IL-1β. These cytokines have a beneficial role in promoting inflammation, but an excessive activation of the inflammasome and the consequent constitutive inflammatory status is a negative factor in human pathologies including Alzheimer’s Disease (AD). MicroRNAs (miR-NAs) target the 3′UTR region of NLRP3, preventing the activation of the inflammasome and inhibiting cytokine production. Because Stavudine (D4T), an antiretroviral drug, was recently shown to reduce inflammasome activation, we verified whether its effect is mediated by miR-7-5p, miR-22-3p, miR-30e-5p and miR-223-3p: miRNAs that bind the *NLRP3*-mRNA-UTR region and interfere with protein translation, reducing NLRP3 activation. Peripheral blood mononuclear cells (PBMCs) of twenty AD patients and ten sex-matched Healthy Controls (HC) were stimulated with Lipopolysaccharides (LPS)+Amyloid-beta (Aβ_42_) in the absence/presence of D4T. Expression of genes within the inflammasome complex and of miRNAs was evaluated by RT-PCR; cytokines and caspase-1 production was measured by ELISA. Results have shown that: NLRP3, ASC, IL-1β and IL-18 expression, as well as IL-18, IL-1β and caspase-1 production, were significantly augmented (*p* < 0.05) in LPS+Aβ_42_-stimulated PBMCs of AD patients compared to HC. D4T reduced the expression of inflammasome genes and cytokine production (*p* < 0.005). miR-7-5p and miR-223-3p expression was significantly increased in LPS+Aβ_42_-stimulated PBMCs of AD patients (*p* < 0.05), and it was reduced by D4T in AD alone. In conclusion: miR-223-3p and mir-7-5p expression is increased in AD, but this does not result in down-regulation of NLRP3 inflammasome expression and of IL-1β and IL-18 production. D4T increased miRNA expression in HC but had an opposite effect in AD, suggesting that miRNA regulatory mechanisms are altered in AD.

## 1. Introduction

Alzheimer’s disease (AD) is associated with impaired cognition and the accumulation of amyloid-β peptides (Aβ) and neurofibrillary T-tau protein tangles in the brain. Neuroinflammation is suggested to play a pivotal role in the pathogenesis of AD [1,2,3], as this disease is associated with activation of microglia and recruitment of peripheral monocytes in the central nervous system (CNS) [4,5,6,7,8,9]. Once in the CNS, monocytes produce inflammatory cytokines as well as molecules with direct neurotoxic effects. Notably, Aβ can directly stimulate the activation of the NLRP3 inflammasome. This results in the assembly of NLRP3 (nucleotide-binding do-main leucine-rich repeat (NLR) and pyrin domain containing receptor 3), ASC (apoptosis-associated speck-like protein containing a caspase recruitment domain), and procaspase- 1 proteins, a process that leads to the cleavage and activation of caspase-1, and the maturation and secretion of IL-1β and IL-18 [10,11,12,13]. 

Different inflammasomes have been shown to be involved in neurodegenerative diseases [14,15,16] but a close association between AD and NLRP3 has been convincingly demonstrated both in animal models of AD [17,18] and in experiments performed using human monocytes [19]. Therefore, pharmacological inhibitors that specifically target the NLRP3 inflammasome could be an option for treatment of AD-associated neuroinflammation. Emerging evidence has shown that Stavudine (D4T), an antiviral nucleoside reverse transcriptase inhibitor (NRTIs) designed to target HIV, down-modulates NLRP3 inflammasome activation as well as IL-18 and caspase-1 production, and stimulates amyloid-β autophagy by macrophages [20,21,22]. Recent results showed that the activation of the NLRP3 inflammasome can be modulated as well by miRNAs, non-coding RNAs that target mRNA and, as a result, reduce transcripts with consequent effect on protein production. miR-7-5p, miR-22-3p, miR-30e and miR-223-3p, in particular, were shown to bind a highly conserved region of the 3′UTR of *NLRP3* mRNA and interfere with protein translation [23], thus reducing NLRP3 activation [24].

Based on the observations that: (1) the NLRP3 inflammasome is a major culprit for inflammation in AD; (2) D4T significantly down-regulates NLRP3 activation; (3) NLRP3 activation is modulated by miRNAs, we analyzed the possible role of 3′UTR of *NLRP3*-binding mRNAs in mediating the hampering effect of D4T on the NLRP3 inflammasome.

## 2. Results 

### 2.1. Clinical Characteristics of the Individuals Enrolled in the Study

Demographic and clinical characteristics of the individuals enrolled in the study are summarized in Table 1. No differences were observed in gender, age, and years of education. As per the inclusion criteria, global cognitive levels (MMSE) were significantly reduced in AD patients (median 21 ± 3.9) compared to healthy controls (>28) (*p* < 0.05). For APOE e-4 carriers, no differences were shown between AD (20%) patients and HC (20%).

With respect to CSF proteins, Aβ (mean 486 ± 109 pg/mL), total-Tau (mean 747 ± 206 pg/mL), and P-Tau (mean 84 ± 44 pg/mL) were analyzed only in patients to confirm the AD diagnosis. Mean values of the CSF protein quantification (Aβ, total-Tau, P-Tau) are shown in Table 1.

### 2.2. MTT Stavudine (D4T)

The MTT assay showed that, in accordance with previous studies [25], PBMC vitality was 90 ± 3.5% using D4T at a 50 µM concentration. 

### 2.3. NLRP3 and Downstream Signaling of Inflammasome Gene Expression in PBMC

mRNA expression of NLRP3, ASC, Caspase-1, IL-18 and IL-1β was quantified by qPCR in cells of all AD patients and healthy controls. Data are expressed as the fold change (n-fold) compared to results obtained in unstimulated cells (medium alone) and those obtained in LPS primed and Aβ_42_ stimulated cells in the absence/presence of D4T. 

NLRP3, ASC, Caspase-1, IL-18 and IL-1β mRNA were significantly up-regulated in LPS+Aβ_42_ stimulated PBMC compared to unstimulated cell (medium alone) PBMC both for AD patients and HC (*p* < 0.05), see Supplementary Material.

**Figure 1:**
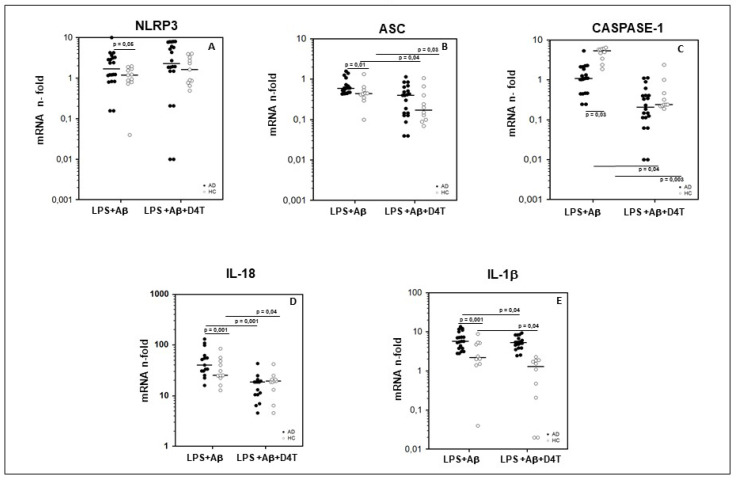
Genes of inflammasome complex. Expression of mRNA for Nod-like receptor protein 3 (NLRP3) (**A**), ASC (**B**), caspase-1 (**C**), IL-18 (**D**), and IL-1β (**E**) is shown. Results were obtained after the samples were primed with lipopolysaccharide (LPS) (1 μg/mL) and stimulated with Aβ42 (10 µg/mL) in the presence/absence of D4T (Stavudine) (50 µM) PBMC of AD (black dots) patients and Healthy Controls (HC) (white dots). Summary results are shown in the dots-plot-graphs and are expressed as the fold change (n-fold) between results obtained in stimulated cells/medium (unstimulated cells). NLRP3, ASC, Caspase-1, IL-18 and IL-1β gene expression was calculated relative to the GAPDH housekeeping gene. Horizontal bars indicate medians. Statistical significance is shown.

**Figure 2:**
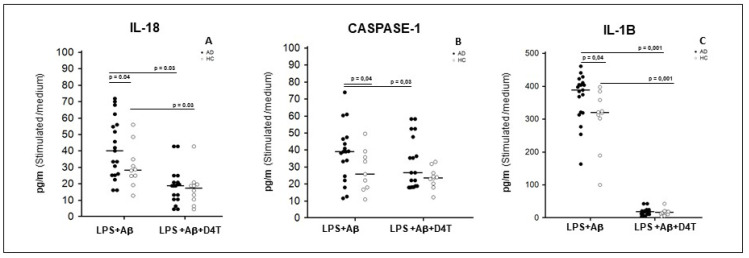
Cytokines and caspase-1 production. IL-18 (**A**), Caspase-1 (**B**), and IL-1β (**C**), production was assessed by multiplex ELISA in supernatants primed with lipopolysaccharide (LPS) (1 μg/mL) and stimulated with Aβ42 (10 µg/mL) in the presence/absence of D4T (Stavudine) (50 µM) PBMC of AD (black dots) patients and Healthy Controls (HC) (white dots). Summary results are shown in the dots-plot-graphs. Summary results are shown in the dots-plot graphs are expressed as the cytokines and caspase-1 production (pg/mL) between results obtained in stimulated cells/medium (unstimulated cells). Horizontal bars indicate medians. Statistical significance is shown.

**Figure 3:**
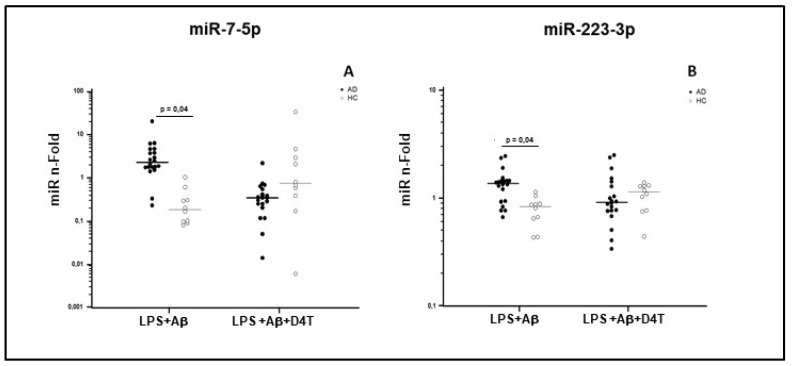
miR-7-5p (**A**) and miR-223-3p (**B**) concentration. Results were obtained after samples were primed with lipopolysaccharide (LPS) (1 μg/mL) and stimulated with Aβ42 (10 µg/mL) in the presence/absence of D4T (Stavudine) (50 µM) PBMC of AD (black dots) patients and Healthy Controls (HC) (white dots). Summary results are shown in the dots-plot-graphs and are expressed as the fold change (n-fold) between results obtained in stimulated cells/medium (unstimulated cells). Row data were normalized relative to a reference miRNA (miR-103-3p). Horizontal bars indicate medians. Statistical significance is shown.

To summarize: (1) NLRP3 (*p* < 0.05) (Figure 1A); (2) ASC (*p* < 0.01) (Figure 1B); (3) IL-18 (*p* < 0.001) (Figure 1D); (4) IL-1β (*p* < 0.001) (Figure 1E) mRNA expression was significantly upregulated in AD compared to HC.

An opposite trend was seen for Caspase-1 mRNA expression, which was upregulated in PBMC of HC compared to AD (*p* = 0.03) (Figure 1C).

D4T addition to cell cultures significantly reduced ASC, Caspase-1, IL-18 and IL-1β mRNA expression in both AD and HC (*p* < 0.05) (Figure 1B–E), and increased that of NLRP3 in AD patients alone (*p* < 0.05) (Figure 1A). 

### 2.4. Inflammasome Related Cytokine Production in Supernatants of PBMC of AD Patients and HC

In in vitro LPS+Aβ_42_-stimulated PBMC, the production of IL-18, IL-1β and caspase-1 (p20 subunit) was significantly increased in AD patients compared to HC (*p* < 0.05) (Figure 2A–C). D4T addition to the culture significantly reduced IL-1β (*p* = 0.001) and IL-18 (*p* = 0.03) production in both AD and HC (Figure 2A–C); caspase-1 production was reduced in HC alone (*p* < 0.05) (Figure 2B). IL-18, IL-1β and caspase-1 were also significantly up-regulated in LPS+Aβ_42_ stimulated PBMC compared to unstimulated PBMC both for of AD patients and HC (*p* < 0.05) (see Supplementary Material).

### 2.5. miRNAs Expression in PBMC of AD Patients and HC

To verify whether the expression of the miRNAs known to bind the 3′UTR region of NLRP3 is different in AD and HC, and whether such expression can be modulated by D4T, we analyzed the expression of miR-7-5p, miR-22-3p, miR-30e-5p, and miR-223-3p in PBMC of AD and HC that were either unstimulated or LPS- and Aβ_42_-stimulated in the absence/presence of D4T. 

Results showed that miR-7-5p and miR-223-3p expression was significantly increased by LPS and Aβ_42_-stimulated PBMC of AD patients compared to HC (*p* < 0.05, Figure 3). Similar results were also observed for miR-30e-5p and for miR-22-3p, although without reaching statistical significance.

Interestingly, D4T treatment increased the expression of miR-7-5p and miR-223-3p in PBMC of HC, but it reduced such expression in AD patients, although without reaching statistical significance (Table 2). Taken together these results indicate an opposite modulation of miR-7-5p and miR-223-3p in PBMC of AD and HC subjects after stimulus with LPS+Aβ_42_ and with LPS+Aβ_4_ +D4T.

## 3. Discussion

A peculiar pattern of miRNA expression was suggested to characterize Alzheimer’s disease [26,27,28,29,30,31], a condition that is also associated with an excessive activation of the NLRP3 inflammasome [17,18,19]. Because the activation of the NLRP3 inflammasome was shown to be modulated by miRNAs [24,31,32], we decided to analyze whether the miRNAs profile seen in AD indicates a role of these non-coding RNAs in NLRP3 activation. Additionally, as D4T, an antiretroviral, was recently shown to inhibit the activation of the NLRP3 inflammasome, we verified if this effect is mediated by the modulation of miRNA expression.

Previous studies have documented that the expression of a number of miRNAs [33], including miR-223 [34,35], is dysregulated in AD and correlates with disease severity, supporting the idea that miR-223 might play a role in the pathogenesis of AD [35,36]. Three additional miRNAs, miR-7-5p, miR-22-3p, and miR-30-5p can bind to the UTR region of NLRP3-mRNA, hampering protein translation and blocking the inflammasome protein complex formation. Finally, other results reported a down-regulation of miR-7 and miR-30e in the brain [32] and a low expression of circulating miRNA-22 in AD patients [37].

In this work, the expression of miR-7-5p, miR-22-3p, miR-30e-5p and miR-223-3p was analyzed in AD patients and compared to that seen in age-matched healthy controls. The ability of D4T to modulate miRNA expression was analyzed as well. Results indicated that miR-223-3p and mir-7-5p are increased in AD patients compared to controls, but this does not result in down-regulation of the NLRP3-inflammasome expression and of IL-1β and IL-18 production. Interestingly, D4T increased miRNA expression in HC but had an opposite effect in AD, suggesting that miRNAs regulatory mechanisms might be altered in AD. Because the most striking differences in miRNA expression were seen when miR-223-3p and miR-7-5p were analyzed, we focused our attention on these two molecules.

miRNAs are powerful and sensitive post-transcriptional regulators of gene expression that directly target messenger RNAs (mRNAs) and inhibit mRNA stability and translation [38]. miR-223-3p, in particular, binds a highly conserved region of the 3′UTR Mrna of NLRP3 and is the best characterized NLRP3 miRNA inhibitor [24,39]. Herein, despite miR-223-3p expression being up-regulated in LPS primed and Aβ_42_-stimulated PBMC of AD patients, no significant reduction was observed in NLRP3 mRNA expression and this resulted in higher caspase-1 cleavage, IL-1β and IL-18 release. Previous experiments in primary human monocytes confirmed that miR-223-3p expression is inversely correlated with NLRP3 levels during macrophage differentiation: miR-223-3p is up-regulated in monocytes and is differently expressed when these cells mature into M1 or M2 macrophages [40]. This miRNA [38] was suggested not to trigger an immediate negative-feedback mechanism, but rather to modulate the priming signals needed to reach a certain “inflammatory level” that leads to the activation of the NLRP3 inflammasome. The paradoxical effect we observed, i.e., significant increase of miR-223-3p without a reduction of NLRP3 activation, could be justified by the use of whole peripheral lympho-monocytes that had not been previously differentiated in monocytes/macrophages, as in previous studies [38,40]. Alternatively, it is possible that the massive NLRP3 expression observed in LPS+ Aβ_42_-stimulated PBMC of AD overrules the inhibitory effect of miR-223-3p on NLRP3 activation, as was previously observed in an in vitro model of endothelial cells that were stimulated with the recombinant proteins of *Treponema pallidum* [41]. 

Our results confirm that D4T, an anti-retroviral drug, reduces NLRP3 inflammasome activation [20], showing a significant dampening effect on the generation of the NLRP3 inflammasome-downstream molecules ASC, Caspase-1, IL-1β and IL-18 mRNA. Interestingly, the effect of Stavudine was different in PBMC of AD patients and HC, as the compound decreased NLRP3 expression in HC but increased it in AD. The increased NLRP3 expression in the presence of a reduction in the concentration of its downstream inflammatory proteins is puzzling. It is known that mRNA could be transcribed without being translated into protein [42,43] and indeed, besides miR-7 5p and miR-223-3p, other miRNAs could be involved in NLRP3-mRNA post-transcriptional regulation; this will need to be further investigated. 

To summarize the results of this pilot study: miR-7-5p and miR-223-3p expression in PBMC was increased in AD patients concomitantly with augmented activation of the NLRP3 inflammasome, and was reduced by D4T. These results suggest that these miRNAs are upregulated in AD in a futile attempt to dampen NLRP3 activation, possibly indicating that the inhibitory effect of these molecules on NLRP3 is lost in AD.

## 4. Materials and Methods

### 4.1. Patients and Controls

Twenty AD patients who fulfilled inclusion criteria for a clinical diagnosis of AD were enrolled from January 2017 to September 2018; these individuals were followed by the Neurology Clinic of Fondazione Cà Granda, IRCCS, Ospedale Maggiore Policlinico in Milan, Italy. The clinical diagnosis of AD was performed according to the NINCDS-ADRDA work group criteria [44] and the DMS IV–R [45]. Neuropsychological evaluation and psychometric assessment were performed with a Neuropsychological Battery [46,47]. All AD patients underwent complete medical and neurological evaluation, routine blood tests, brain MRI, and lumbar puncture (LP) for quantification of the CSF biomarkers Aβ, total tau (tau), and tau phosphorylated at position 181 (Ptau) [48]. 

Ten healthy sex and age matched healthy controls (HC) were also enrolled in the study; these individuals were volunteers without a family history of dementia or evidence of acute or chronic neurologic diseases at the time of enrollment, and were selected ac-cording to the SENIEUR protocol for immuno-gerontological studies of European Com-munity’s Control Action Programme on Aging [49]. The cognitive status of HC was assessed by MMSE (score for inclusion as normal control subjects ≥ 30). APOE genotyping, was available for all subjects and was determined by allelic discrimination [50]. The current study was approved by the Institutional Review Board of the Fondazione Cà Granda, IRCCS Ospedale Maggiore Policlinico (Milan, Italy) and conformed to the ethical principles of the Helsinki Declaration. All patients (or their legal guardians) and controls gave their written informed consent before entering the study. Epidemiological and clinical characterization of patients and controls is presented in Table 1.

### 4.2. Blood Sample Collection and Cell Separation

Whole blood was collected in vacutainer tubes containing ethylenediaminetet-raacetic acid (EDTA) (Becton Dickinson & Co., Rutherford, NJ, USA). Peripheral blood mononuclear cells (PBMC) were separated on lympholyte separation medium (Cedarlane, Hornby, Ontario, CA) and washed twice in PBS at 1500 RPM for 10 min; viable leukocytes were determined using a TC_20_ Automated Cell Counter (Biorad Hercules, CA, USA). 

### 4.3. Cell Cultures

PBMC (3 × 10^6^/mL) were cultured in RPMI 1640 supplemented with 10% human serum, 2 mM L- glutamine, and 1% penicillin (Invitrogen, Ltd., Paisley, UK), incubated with lipopolysaccharide (LPS) (1 µg/mL) (Sigma-Aldrich, St. Louis, MO, USA) for 2 h, and then stimulated with Aβ_42_ (10 µg/mL Sigma-Aldrich, St. Louis, MO, USA) for 22 h in the absence/presence of D4T (50 µM) (Sigma-Aldrich) [25] at 37 °C in a humidified 5% CO_2_ atmosphere. Supernatants and PBMC pellets were collected at the end of the culture period.

### 4.4. D4T Cytotoxicity Assay

Cell toxicity of D4T was measured using MTT cell viability assay as previously described [51]. The 3-(4,5-Dimethylthiazol-2-yl)-2,5-diphenyltetrazolium bromide (MTT; Sigma-Aldrich St. Louis, MO, USA) powder was dissolved in PBS at the concentration of 5 mg/mL. PBMC cells were plated in 96-well plates at an initial density of 1×10^5^ cells/well and were treated with 50 μM concentration of D4T [25]. Cells were incubated at 37 °C for 22 h and centrifuged; pellets were dissolved using 100 μL/well of dimethyl sulfoxide (DMSO), and plates were read in a microplate reader using a test wavelength of 550 nm and a reference wavelength of 650 nm. Results were calculated as follows: % cytotoxicity = 100 − (OD test − OD control)/OD control × 100. Cell mortality was comparable (<5%) to unstimulated condition.

### 4.5. Total RNA Extraction

Total RNA was extracted from 3 × 10^6^ unstimulated or stimulated (see above) PBMCs using a column-based kit (miRNeasy Mini Kit, Qiagen GmbH, Hilden, Germany cat. 217004) according to the manufacturer’s protocol. RNA concentration was determined by a spectrophotometer (Nanoview plusTM, GE Healthcare, Little Chalfont, UK). Purity was determined as the 260/280 nm OD ratio, with the expected values between 1.8 and 2.0. RNA was treated with TURBO DNA-free DNAse (Ambion Inc., Austin, TX, USA).

### 4.6. NLRP3-Inflammasome Pathway Quantitative Transcriptional Analysis by Real Time PCR

A portion of the extracted-RNA (20 ng) was reverse-transcribed in cDNA using RT2 First Strand Kit (Qiagen, Hilden Germany) according to the manufacturer’s protocol. All NLRP3 (cat. PPH13170A), ASC (cat. PPH00907A), caspase-1 (cat.PPH00105C), IL-1β (cat. PPH72244A), and IL-18 (cat.PPH00580C) (Qiagen) primers were cDNA specific. Quantitative real-time RT-PCR (qPCR) was performed using a Biorad CFX Real-Time PCR instrument. mRNA expression of NLRP3, ASC, Caspase-1, IL-18 and IL-1β was analyzed using RT2 SYBR Green qPCR mastermix (Qiagen, Hilden Germany). Results are expressed as Ct and presented as the ratio between the target gene and the glyceraldehyde 3-phosphate dehydrogenase (GAPDH) (Qiagen) (cat. PPH68912A) housekeeping mRNA. Experiments were individually run on each one of the individuals included in the study.

### 4.7. miRNA Quantitative Analysis by Real Time PCR

Twenty nanograms of the extracted-RNA were retrotranscribed into cDNA using the universal cDNA synthesis kit (miRCURY LNA Universal cDNA synthesis kit, Qiagen, Hilden, Germany), according to the manufacturer’s protocol. Specific LNATM-individual microRNA assays (Qiagen, Hilden, Germany) were utilized to quantify hsa-miR-7-5p (cat.YP00205877), hsa-miR-22-3p (cat. YP00204606), hsa-miR-30e-5p (cat. YP00204714) and hsa-miR-223-3p (cat. YP00205986); reference miRNA (hsa-miR-103a, cat.204063) (Qiagen) was used to normalize the results. qPCR was performed using a real time PCR system (CFX96Touch real-time PCR Detection System, BioRad, Hercules, CA, USA) in 10 μL of reaction mix containing SYBR GREEN master mix (Exiqon Inc., Qiagen, Hilden, Germany), a specific primer set for each miRNA, and 4 μL of cDNA (30 × diluted). Each cDNA template was tested in triplicate. Negative controls, without rt-template controls, as well as no-template controls were included in each session. An additional step in the qPCR analysis was performed to evaluate the specificity of the amplification products by generating a melting curve for each reaction. For each sample, relative gene expression of the target miRNA was calculated as the ratio between the target gene and the endogenous reference miRNA [52] using the qBase+ software (version 3.0, Biogazelle, Belgium). Changes in miRNA expression (nFold) were calculated relative to unstimulated PBMC. miRNA relative expression was analyzed after logarithmic transformation.

### 4.8. Elisa

Concentrations of IL-1β, IL-18 and Caspase-1 in the supernatants of unstimulated and stimulated (see above) PBMC were analyzed by sandwich immunoassays according to the manufacturer’s instructions (Quantikine Immunoassay; R&D Systems, Minneapolis, MN, USA). A plate reader (Sunrise, Tecan, Mannedorf, Switzerland) was used and optical densities (OD) were determined at 450/620 nm. Sensitivity (S) and Assay Range (AR) were as follows: S: IL-1 = 1pg/mL; Caspase-1 = 1.24 pg/mL; IL-18 = 12.5 pg/mL. AR: IL-1 3.9–250 pg/mL; Caspase-1 = 6.3–400 pg/mL; IL-18 = 25.6–1000 pg/mL.

### 4.9. Statistical Analysis

Data analysis was performed using the MedCalc statistical package (MedCalc Soft-ware bvba, Mariakerke Belgium). For mRNA and miRNA expression, which was not normally distributed, the Mann–Whitney test was used to compare the fold change value in AD and HC (unpaired samples), while the Wilcoxon test was used to compare different stimuli (LPS+Aβ_42_ vs. LPS+ Aβ_42_ +D4T) within AD and HC (paired samples). For cytokine production, comparisons between AD and HC were based on the non-parametric Mann–Whitney test because the sample size was too small (20 AD and 10 HC) and the probability of normality test was greater than 0.05. All of the results are summarized as fold-change expression from the unstimulated samples. Summary results are shown in the dots-plot graphs. Horizontal bars indicate medians. *p*-Values of less than 0.05 were considered statistically significant.

## Figures and Tables

**Table 1:** Demographic, clinical and genetic characteristics of the individuals enrolled in the study.

	AD	HC
N	20	10
Gender (M:F)	05:05	04:06
Age (years)	76 ^a^ ± 5.9 ^b^	72 ^a^ ± 6.2 ^b^
Level of education (years)	8.25 ^a^ ± 2.71 ^b^	7.62 ^a^ ± 3.62 ^b^
MMSE (Baseline)	21 ^a^ ± 2.9 ^b^	>28
APOE e-4 cariers (%)	20 ^a^	20 ^a^
Amyloid-β (pg/mL)	486 ^a^ ± 109 ^b^	_
Total-tau (pg/mL)	747 ^a^ ± 206 ^b^	_
P-tau (pg/mL)	84 ^a^ ± 44 ^b^	_

Data are expressed as mean (^a^) ± standard deviation (^b^). MMSE: Mini-Mental State Examination.

**Table 2:** Summary results of miRNA expression in PBMC of AD patients and HC.

		AD			HC		
	Condition	*N*	Median	IQR	*N*	Median	IQR
miR-7	LPS+Aβ	20	2.170	1.625–4.455	10	0.100	0.083–0.823
	LPS+Aβ+D4T		0.355	0.250–0.640		0.810	0.225–4.185
miR-223	LPS+Aβ	20	1.424	0.964–1.511	10	0.865	0.659–0.920
	LPS+Aβ+D4T		0.993	0.737–1.396		1.087	0.755–1.281

## Data Availability

Data is contained within the article.

## Supplementary Materials

The supplementary materials are available online at www.mdpi.com/article/10.3390/ph14111187/s1.
