# Supplementary material for: Pharmacological and Epigenetic Regulators of NLRP3 Inflammasome Activation in Alzheimer’s Disease
Source: Pharmaceuticals (Basel). 2021 Nov 20;14(11):1187. doi: 10.3390/ph14111187 (PMC8623160; doi:10.3390/ph14111187)
Supplement: Supplementary file 1 [file pharmaceuticals-14-01187-s001.zip › pharmaceuticals-1425127-supplementary.pdf]

Supplementary data

	AD			HC		
**mRNA expression** *(n-fold)*	**N**	**Median**	**IQR**	**N**	**Median**	**IQR**
	**20**			**10**		
**Nlrp3**	Med	0,25	0,021 - 0,882	Med	0,23	0,032 - 0,782
	LPS+Aβ	2,09*	1,177 - 3,183	LPS+Aβ	1,065*	0,880 - 1,540
**ASC**	Med	0.12	0,051 - 0,962	Med	0,45	0,403 - 0,662
	LPS+Aβ	0,69*	0,462 - 0,768	LPS+Aβ	0,95*	0,600 - 0,970
**Caspase-1**	Med	1,09	0,470 - 2,025	Med	5,41	3,430 - 5,970
	LPS+Aβ	2,29*	0,890 - 2,325	LPS+Aβ	19,35*	17,620 - 25,37
**IL-1β**	Med	5,71	3,765 - 8,709	Med	0,56	0,400 - 1,040
	LPS+Aβ	15,91*	13,265 - 21,809	LPS+Aβ	2,205*	1,500 - 5,240
**IL-18**	Med	12,008	11,143 - 15,891	Med	2,24	1,163 - 2,892
	LPS+Aβ	55,52*	51,365 - 58,256	LPS+Aβ	14,56*	12,863 - 18,682

	AD			HC		
**Protein production (pg/ml)**	**N**	**Median**	**IQR**	**N**	**Median**	**IQR**
	**20**			**10**		
	Med	9.69	8,910 - 12,890	Med	11.58	11,910 - 18,890
**Caspase-1**	LPS+Aβ	39.04*	29,884 - 38,321	LPS+Aβ	35.72*	24,585 - 36,541
	Med	4.99	12,980 - 16,230	Med	6.65	3,980 - 5,230
**IL-1⚜**	LPS+Aβ	388.65*	319,420 - 409,187	LPS+Aβ	328.00*	309,420 - 400,187
	Med	7.08	2,980 - 6,230	Med	3.05	2,980 - 6,230
**IL-18**	LPS+Aβ	53.36*	11,910 - 21,890	LPS+Aβ	33.65*	26,884 - 35,321

MED = UNSTIMULATED CELL (MEDIUM ALONE); AD= ALZHEIMER'S DISEASE PATIENTS; HC= HEALTHY CONTROL; *= $p < 0.05$
